# Patient and provider perspectives on implementing patient navigation for colorectal cancer screening for Black and Latine patients: a qualitative study

**DOI:** 10.1186/s12913-025-13185-8

**Published:** 2025-07-28

**Authors:** Katarina E. AuBuchon, Laura C. Schubel, Jessica N. Rivera Rivera, Demetrie Garner, Jennifer Tran, Sophia Urdinola, Hannah Arem

**Affiliations:** 1 Lombardi Comprehensive Cancer Center, Georgetown University, Washington, DC USA; 2 Implementation Science, Healthcare Delivery Research Network, MedStar Health Research Institute, Washington, DC USA; 3 Department of Medicine, MedStar Washington Hospital Center, Washington, DC USA; 4 Department of Oncology, Georgetown University, Washington, DC USA

**Keywords:** Colorectal cancer, Patient navigation, Cancer screening, Health inequities, Qualitative, Multilevel interventions

## Abstract

**Background:**

In the United States, Black people experience inequities in colorectal cancer (CRC) screening access, contributing to CRC outcome inequities. Latine people in the US and have lower screening rates (53.4% vs. 70.4% for White people), and CRC is the leading cause of all cancer death among this population. Patient navigation is an evidence-based approach to increase CRC screening, however it is not often implemented at scale. We interviewed patients and providers about barriers and facilitators to CRC screening and scaling a patient navigation program for Black and Latine patients in a mid-Atlantic healthcare system.

**Methods:**

We interviewed screening-eligible (age 45–75) patients (*n* = 15; 46.7% Black, 53.3% Latine) and healthcare system partners (*n* = 12; 42% primary care, 33% gastroenterology, and 25% systems-level administration). Interviews were in Spanish and English, and responses were analyzed qualitatively with a pragmatic thematic analysis to inform program implementation.

**Results:**

Nearly all patients and partners identified that CRC education and screening education were barriers to timely screening, and identified navigators as education brokers. Patients expressed that education on stool tests and colonoscopies is an essential part of informed decision-making, and can be facilitated by navigators. Navigators can also provide support for addressing or overcoming emotional or practice barriers. Navigators are further uniquely positioned to foster a trusting relationship through clear, direct, and timely communication with patients. Healthcare system partners suggested that navigators assist in identifying patients in need of CRC screening and facilitating closed-loop communication about screening completion. Anticipated barriers to implementation of a patient navigation program included buy-in from primary care providers and clinical administrators.

**Conclusions:**

Implementing CRC navigation was perceived as a potential solution to multilevel barriers to CRC completion for Black and Latine patients. Future work may consider identifying effective implementation strategies to ensure maximum navigation reach and effectiveness.

## Background

Historically marginalized racial and ethnic populations experience higher mortality from colorectal cancer (CRC) than White populations. From 2016 to 2020 in Washington, District of Columbia (DC), the CRC mortality rate was 2.7 times higher for non-Hispanic Black (hereafter Black) vs. non-Hispanic White people, and in Maryland the CRC mortality rate was 1.3 times higher for non-Hispanic Black people compared to non-Hispanic White [1]. Half of the disparity in outcomes for Black individuals is attributed to lower screening access [2]. CRC is a leading cause of cancer death among Latine (people who identify as Hispanic, Latina, Latino, Latinx, etc.) individuals in the United States, accounting for 12% of deaths among men and 9% among women [3]. 

CRC screening is the definitive, evidence-based approach to reduce morbidity and mortality from CRC [4, 5], but there are significant disparities in screening rates among some historically marginalized populations. In national surveys, Latine individuals have the lowest screening rates out of aggregated racial/ethnic groups, with a 2021 average of 61.7% up to date with screening compared to 74.6% screened among White individuals [6, 7]. Previous studies have cited barriers to colonoscopy completion among Black and Latine individuals including lack of knowledge, fear/anxiety surrounding the procedure (e.g., sexual stigma, fear of pain, fear of cancer diagnosis), and lack of provider communication about CRC and screening options [8, 9]. Limited provider interviews have suggested logistical barriers around cost are important to consider, in addition to some of the patient-identified barriers [10]. 

There is a plethora of evidence to support the effectiveness of patient navigation in reducing disparities in CRC screening. Patient navigation includes assessing a patient’s individual barriers such as transportation, stigma, fear, finances, or trouble preparing, and helping the patient to overcome identified screening barriers, and can be directed by community health workers (i.e., trained frontline health workers) [11]. One systematic review of 76 studies that used community health worker navigators demonstrated a median increase of 10.5% points in colorectal cancer screening after navigation intervention regardless of race or ethnicity, income, or insurance status [12]. Despite this evidence for patient navigation to reduce CRC screening disparities, it is not being delivered at scale. In this study, we examined patient and healthcare system partners’ (e.g., clinicians, practice managers, operations/administrative staff) barriers to CRC screening completion among Black and Latine patients in our catchment area, and the role of navigation in addressing CRC screening barriers. Additionally, we identified barriers to implementing navigation at scale in primary care practices using contextual elements from the Practical, Robust Implementation and Sustainability Model (PRISM) to frame important questions about patients and organizational characteristics and perspectives [13, 14]. 

## Methods

The present work is a part of a larger, stepped-wedge cluster randomized controlled trial to assess implementation of patient navigation for CRC screening [15]. 

### Participants and healthcare system setting

In conjunction with our clinical co-investigators, we identified a sample of 11 Primary Care (PC) clinics within our mid-Atlantic healthcare system. We chose the 11 clinics to include distinct geographic locations (urban vs. suburban, Maryland (MD) vs. DC) in the system’s catchment area and patient populations with diverse characteristics such as preferred language (oversampling Spanish speakers) and payor mix. We considered a greater or smaller number of clinics but ultimately decided that the proposed sample covered our needs regarding variability in patient and geographic characteristics.

This research was performed in line with the principles of the Declaration of Helsinki and was approved by the MedStar-Georgetown institutional review board (IRB# 6963). We requested permission from medical directors prior to reaching out to patients for interviews. For healthcare system partners, we recruited those who had a centralized healthcare system role in PC or Gastro-intestinal (GI) clinics, as well as to practice managers or providers who had experience trying to implement CRC screening programs, balancing recruitment across the two major catchment areas in MD and DC. Additionally, we used a snowball sampling strategy where healthcare partners also suggested other system partners as potential participants. At the time of the study, there was no existing centralized patient navigation program for CRC screening, but there were some localized programs within specific clinics (that were excluded from the parent study to implement navigation, but not from formative interviews).

Patient participants were recruited via phone using purposive selection from generated lists from the Electronic Health Record (EHR) of those age 45–75 who identified as Black or Latine and had a primary care visit within the prior year. Potential patient participants were classified as due for CRC screening if the EHR indicated they were due. While we used EHR data to try to identify participants due for screening, during the interviews many participants told us they had completed screening, and were still retained in our study. Healthcare system partner (hereafter referred to as partners) participants had roles in PC or gastroenterology (GI) specialty clinics and were involved in administration or delivery of referrals for CRC screening or had been engaged in other navigation programs. Participants were recruited via phone or email, and interviews were conducted via phone or video chat. All participants received a written copy of the informed consent via email and provided verbal consent prior to the interview.

### Qualitative procedure and analysis

A standardized semi-structured interview guide (Table 1) based on PRISM contextual factors, as well as patient and organizational characteristics and perspectives was developed to inform a planned patient navigation intervention in PC [16]. The patient interview guide specifically built on existing literature on barriers to CRC screening among Black and Latine community members, including knowledge of screening, barriers and facilitators, stigma or experiences of discrimination, and thoughts or preferences on the proposed patient navigation program. The healthcare system partner interview guide focused on similar patient-level topics, but also strategies for communicating with participating practices and practice-level characteristics and perspectives around implementing a navigation program into existing workflows. Our study’s community advisory board, described elsewhere [15], provided feedback on both interview guides. In the fall of 2023, KA, LS, HA, and JRR conducted one-hour interviews with participants via Microsoft Teams in English and JRR (native Spanish speaker) conducted the interviews in Spanish. Interviewers explained the purpose of the study and obtained verbal consent. Results were summarized in a structured table to capture the main themes. At weekly meetings, the team discussed results and data saturation. Both English and Spanish interviews were recorded and transcribed automatically before interviewers conducted quality control to ensure accurate capture of language and to edit interviews for clarity (e.g., eliminating repeated words or “umm”). One interview was not recorded due to patient preference, and detailed notes were taken instead by the interviewer. Interviews in Spanish were reviewed for quality by JRR and SU (native Spanish speakers). All transcripts were uploaded into Dedoose for analysis.

**Table 1:** Example interview questions for providers and patients

Domains	Example Questions	
	Patient	Provider
CRC screening options	Have you ever been screened for CRC? What was your experience like? [After explaining about stool tests and colonoscopies] Which of these tests would you prefer to get? Tell me more about how you made that decision.	Can you describe what is currently being done to facilitate CRC screening in your clinic/environment? What is the current process for making different CRC screening options available to patients?
Barriers and facilitators to CRC screening	What things might make it hard for you or someone like you to get screened for CRC? What do you think doctors should do to encourage CRC screening for you or your friends and family? Colonoscopies are invasive, meaning they can only be done inside of the body. To some people, the invasiveness can be a barrier to screening because they think colonoscopies are somehow related to sex or masculinity or are uncomfortable with the fact that the procedure involves a sensitive body part. You may feel this way or you may not, and either way is okay. What are your thoughts on this issue?	What barriers to CRC screening do you think are most important and impactful to your patients? In what ways can clinics and staff address these barriers? What are the biggest issues with referrals to GI clinics for colonoscopy or to receiving FIT/Cologuard?
Patient navigation implementation and tailoring	What kinds of things can patient navigators say or do to help people get screened for CRC? If a patient navigator was helping you set up CRC screening, what would you like help with?	What are your impressions of a patient navigation program providing remotely delivered supports for patients to receive CRC screening?
Addressing systemic racism and mistrust	How do you think [experiencing discrimination] could make people feel differently about getting CRC screening? What kinds of things may get in the way for a [Black man, Black woman, Latina, Latino] to get CRC screening? What may make it easier?	If you could offer any program to support Black and Latina/o patients to complete CRC screening, what would it look like? What are the complexities that you can think of with offering phone-based colorectal cancer screening navigation?

We took a pragmatic qualitative approach using narrative thematic analysis [16]. Thus, we created a-priori codebooks (one for patient interviews, one for partner interviews) to answer specific questions about implementing the navigation program for the parent study. Four independent coders (KA, LS, JRR, and DG) met to discuss each codebook and create operational definitions for each code. All coders (KA, LS, JRR, DG) coded one interview to start (patient or healthcare partner) and the codebooks were edited iteratively based on team feedback. The team coded three patient and two healthcare partner interviews simultaneously to reach at least 80% coding agreement with KA. After agreement was reached (3 interviews coded), the team members independently coded the remaining interviews with weekly meetings to discuss inconsistencies, disagreements, and coding questions, which were resolved through discussion until consensus was reached. Representative quotes were selected from the identified themes. Our reporting is consistent with the Consolidated Criteria for Reporting Qualitative Research (COREQ) guidelines [17]. 

### Reflexivity

Reflexivity is a continuous process through which researchers self-reflect on the role of lived experiences and assumptions brought to the research process and products [18, 19]. We considered personal, interpersonal, methodological, and contextual influences on this work [20]. In our personal and interpersonal reflexive process, we acknowledged our roles in shaping the conclusions we drew from these interviews [21]. During drafting of interviews and prior to interview start, the research team discussed positionality as a role in influencing the interviews and analysis process. Team members were encouraged to memo, review, and reflect on our backgrounds and professional positions as influences on the interviews and analysis.

## Results

We conducted a total of 15 patient and 12 healthcare system partner interviews. We called 102 patients, of whom 61 (61.6%) did not respond to outreach, 21 (21.2%) declined to participate (no reason provided, scheduling conflicts), and 5 (5.1%) were lost to follow-up (i.e., non-response when called at a scheduled interview time). Interviewee demographics are in Table 2. Patients were on average 57.6 years old (SD = 9.7; median = 55, range 48–75) and approximately half (*n* = 7; 46.7%) identified as non-Hispanic Black, and Latine (*n* = 7; 46.7%); one participant identified as Black and Latine (6.7%). All but one patient had some form of health insurance (*n* = 14; 93.3%), and eight (53.3%) participants were not born in the United States. All 12 healthcare partners we approached agreed to participate. Healthcare system partners on average were in their current roles 8.2 years (SD = 7.4) and half (*n* = 6) were physicians. All had higher than a college degree and half (*n* = 6) of the sample identified as White. Eight participants *n* = 8 (66.7%) identified as women.

**Table 2:** Demographics

	System Partners (*N* = 12)		Patient (*N* = 15)	
	*N*	%	*N*	%
Highest level of education				
Less than high school	-	-	4	26.7
High school	-	-	2	13.3
Some college	-	-	1	6.7
Associates degree	-	-	1	6.7
College degree	1	8.3	5	33.3
Graduate degree	5	41.7	2	13.3
Doctoral degree	6	50.0	0	0.0
Ethnicity				
Of Hispanic or Latino origin	2	16.7	8	53.3
Not of Hispanic or Latino origin	3	25.0	7	46.7
Racial Group				
Asian	2	16.7	0	0.0
Black/African American	3	25.0	8	53.3
White	6	50.0	1	6.7
Other	0	0.0	6	40.0
Prefer not to answer	1	8.3	0	0.0
Gender identity				
Woman	8	66.7	8	53.3
Man	3	25.0	7	46.7
Prefer not to answer	1	8.3	0	0.0
Role				
Physician (MD/DO)	6	50.0	-	-
Advanced Practice Practitioner (NP)	1	8.3	-	-
Operations (corporate or related)	3	25.0	-	-
Practice Manager/Clinic Supervisor	2	16.7	-	-

Patients did not have medical positions and seven system partners did not answer the question regarding their ethnicity

### Patient-level barriers to CRC screening

#### CRC knowledge and education

Table 3 summarizes patient-level barriers to CRC screening identified in the interviews. Nearly all patients (*n* = 13; 86.7%) had completed some kind of CRC screening in the past, yet participants recounted not knowing about CRC screening or the disease, nor understanding what the colon is. Some participants at first told the interviewer that they had not received CRC screening, before they recounted that they had received one after the interviewer described a colonoscopy. One participant told the interviewer that he thought his colonoscopy and Cologuard were forms of prostate cancer screening and was confused to find out that it was CRC screening.

**Table 3:** Patient-level barriers and navigation solutions

	Patient Quotes	Healthcare system partner quotes
CRC Knowledge & Education	P14 (57-year-old English-speaking Latino) “Lack of information and lack of education. […] ***I don’t feel that there’s enough information about colon cancer***.” P11 (54-year-old Black man) *Participant*: Well, I talked to my doctor about it.I think I did the prostate cologuard kit? *Interviewer*: Yeah, that is colon cancer screening. *Participant*: So it’s going to take two weeks [to get my Cologuard results back]. So I done that now my next step is when I go see my primary care, tell him I want to do my prostate. That’s the camera right? *Interviewer*: No, the camera is a colonoscopy. So that’s the colonoscopy. Prostate is different. *Participant*: Was that? How did you do prostate? I thought I did prostate? *Interviewer*: I think prostate- I’m less in the know about it, but I’m pretty sure that prostate is a blood sample. It’s called a prostate antigen test. So it’s different. *Participant*: Wait a minute, I think I’m confused.” P10 (59-year-old English-speaking Black man) “Yeah, and see that’s another thing. I didn’t know about cologuard! And I didn’t know until my wife got her box delivered to the house” P9 (58-year-old English-speaking Black woman) “For you to be able to choose which one you feel is best for you really entices a person to do it because they feel like it’s their choice. You’re not making that choice for me, I’m making the best choice for myself. […] ***You give them the options and let them choose and they want to choose what’s best for them.”*** P8 (50-year-old Spanish speaking Latino) “You know how to motivate them ***because if no one has***,*** no one has***,*** no one has information***,*** [sic] no one can spread the information***.” P16 (English-speaking Black woman unknown age) “Because I think the colonoscopy itself, ***my husband [sic] said it was uncomfortable and stuff. So I think I like the stool sample better*** (laughs). [interviewer asks to clarify if she means the preparation] Yes and liquid that they drink and how they go about testing it after you drink it.”	PR12 (GI provider) “***Just try to assess in clinic their health literacy***,*** like how much education do they need to prepare for [a colonoscopy]***. Cause a lot of patients are completely baffled by it. Or just the prized and feel. It’s very invasive. Sort of getting over that barrier or just educating them. And then so you end up spending, you know, your 30-minute visit predominantly educating patients on the prep” PR4 (PC provider) “I mean, I know that we, we did have like signs that say, it’s colorectal cancer screening month, things like that to try to encourage people to have it done. I’m not really sure what [other] providers talk to the patients about in the rooms, but I’m sure that they do provide the education that ***they need to try to explain to them how important it is to have this done***.” PR6 (GI Provider) “And ***I think the most important part is the education***. And patient I don’t think a lot of times ask questions. […] So even if a patient meets with our provider, sometimes they’ll come out and ask our checkout person. Yeah, the doctor told me this, this this, but I you know, I didn’t want to ask that question. What did they mean by that? And we say, Oh, you don’t let’s go talk to them again and find out even if we know the answer, it’s just okay to talk to the doctor. And I think some of that may just be cultural.”
Language & Literacy	P2 (50-year-old Spanish speaking Latina) “No, for me ***[the words you use] doesn’t matter***. No, they’re not going to offend me [by using words like “poop,” “rectum,” etc.], they’re not going to tell me that I’m from the street or anything. That’s what they’re talking to me about things for my own good.”	Provider 8 (Operations Staff) “So I would say the population that we’re targeting here, probably the Hispanic population, [have trouble] ***because they don’t understand the prep instructions. So we do have a Spanish version*** [of the prep instructions]. So that, I think is just making sure that whoever’s the navigator, we team up with this person to [make sure they are able to explain the instructions] in Spanish very thoroughly.”
Colonoscopy Logistics	Patient 2 (50-year-old Spanish-speaking Latina) “They have to go to the doctor, whether it’s work, not much work, maybe they don’t have insurance, that’s the main thing, many people don’t have insurance, they’re afraid, oh, ***How am I going to do that if it’s expensive and I don’t have insurance?*** Most people don’t have any [insurance], see.” Patient 9 (58-year-old English-speaking Black woman) “***Because like when you go get a colonoscopy***,*** you’re off work for that day.*** So you spend a whole day off work. So that might be a deterrent too because you know, you can’t go back to work from that, like a mammogram, you can just walk in there, and they will put your breast upon the thing, do what they gotta do, and you go right back to your job. ***But colonoscopy***,*** you can’t do that you have to give a whole day of work***.”	Provider 7 (PC physician) “Cost if they’re worried about a, either the copay, like I actually had someone who said that they were told they had to put X amount of money up front. First, before they would even get scheduled for their colonoscopy. You know what I mean? ***So right there that that’s an immense barrier to say***,*** I’m going to do something unpleasant to you***,*** but you’re gonna have to pay me $200 upfront for me to even think about it.***” Provider 4 (PC physician) “***I would say definitely***,*** you know***,*** the insurance***,*** of course***,*** it’s always an issue.*** Does your insurance cover it? […] Transportation is definitely a big issue here. I mean, with a colonoscopy, it’s not just getting to the appointment and having to get away at home as well. […] ***And then not the pre op testing***,*** prior to having a colonoscopy as another visit to a doctor’s office***, before even having a procedure done.”
Fear of colonoscopy or cancer	P16 (58-year-old English-speaking Black woman) “The thing is they don’t get their colon checked or and/or they don’t think about having their colon checked. ***Cause you know a lot of people don’t even go to the doctor***,*** they fear going to the doctor’s office.”*** Pt.7 (45-year-old English-speaking Latina) “***Well***,*** if I would have a [strong family] history***,*** I will actually will be afraid to do it.*** I know some people that could be the reason why they will do it. But that could be one thing that will hold me back, you know? Surely that will hold me back a little bit.” Pt 2 (50-year-old Spanish-speaking Latina): “Well, my thought ***is that there are people who someone talks to them about the cancer screening they should do***,*** and they prefer to stay hidden***,*** not having a test***,*** and don’t do anything***. And then, oh, no, I don’t want to do that, what if they tell me I have cancer. Those are people who are hidden because if they tell you that you have cancer, you have to be up to date with that medication. If you are in time [with cancer diagnosis], you are in time, but if you are not in time, you know that you are not in time because you were stupid, you are not in time. […] Yes, they are terrified of that [finding out that they have cancer], and they shouldn’t be.” Patient 14 (57-year-old English-speaking Latino) “It would be very helpful [if patients] ***had the option of having a family member***,*** be part of one of the appointments so that the patient can feel like***,*** supported***, so it doesn’t become taboo to talk about it. [So] that patient doesn’t have to go back to the home and have to discuss it with the family, because they may leave [some things] out being that they don’t feel comfortable about it. So having a family member as a support at one of the appointments […] they can hear about it, they can understand about it, and they can talk about it.”	Provider 9 (PC physician) “But some of them ***it’s like they had a family member this was for breast cancer***,*** but they had a family member die of breast cancer. And they don’t want to know if they have cancer. Like they’d rather die than know***,*** because it’s like the fear.*** [sic] Second thing is, and this on the patient side that the patient is like reluctant because they remembered, you know, their neighbor told them a story about like, a bad prep experience.”
Masculinity & Sexuality	P14 (57 year old English-speaking Latino) Well, ***I do know***,*** for a fact as a gay man how Latino men can be about anything that has to do with anal sex or anybody even touching them on their back side***,*** per se. It’s like***,*** ‘no***,*** no***,*** no***,*** me never***,***’ you know?*** And it’s, it’s interesting, because you’d have to explain to them you know, doesn’t make you gay to get your anus checked out. It seems like there’s no middle. [They] just say [men are only either] hyper masculine, macho, or, almost submissive and feminine. P10 (59 year-old English-speaking Black man) Like for example, my cousin […] He asked me about when I went to the doctor, and they put me under, sticking the camera up there in your backside. ***He was like***,*** Man***,*** I ain’t – no! – ain’t nobody putting that in my backside man!*** [Laughs]” P5 (52 year-old Black man) Well, when I told people that I was getting my colonoscopy, I think somebody mentioned that, ***oh***,*** you have something stuffed in your butt.*** You know, all that stuff? […] because you know […] ***when somebody is going to see your butt***,*** [it’s] kind of like***,*** gay***,*** you know?*** People teasing, you know, […] ***like ain’t nobody sticking something up my butt.****”*	Provider 10 GI physician “I will say, you know, I still get surprised. You know, 20 years in, that ***I do hear my male patients saying frequently***,*** you know***,*** I don’t want anything in my butt.*** You know, it’s like this is not a sexual it has no sexual connotations whatsoever. And I swear I hear it at least once a month ***from a male patient ‘I’m glad you’re a woman***,*** I wouldn’t have any male doctor going in my butt.’*** I hear, I mean these are things that you know are really thinking about, you know, my own personal bias is that you know, a lot of women who’ve born children and had children. I mean, privacy is off the table. Once you become a mother, you know the whole labor and delivery room. So maybe there’s slightly less reticence on the part of women. Just again, just anecdotally see some differences in how men will approach colonoscopy-based screening.”

Emphasis added to quotes

Around half of patients reported a lack of awareness that there are multiple CRC screening options. Patients stated that learning about the different screening methods (e.g., stool tests) can facilitate informed decision-making about CRC screening and encourage individuals who fear colonoscopies to complete a stool test. PC clinicians also identified a gap in patient education about CRC screening, its importance, and educating patients on screening options.

Participants further discussed ways that patient navigators could help overcome knowledge gaps. Patient participants suggested open conversations between providers and patients about why CRC screening is important, and education on what the colon is and where it is located. Patients further emphasized that navigators may build trust with patients through direct communication, and suggested videos about the screening procedure for education. Some patients expressed that women may perceive they have lower CRC risk than men, suggesting navigators provide education about the importance of CRC screening regardless of sex.

Both patient and partner participants affirmed the importance of Spanish-speaking navigators, even when a translator might be available. Partners also discussed other language services needed for our catchment area (e.g., Amharic). Clinicians discussed how language barriers make patient education more difficult, and that it can impact patients’ colonoscopy preparation.

Some GI partners identified colonoscopy preparation (prep) as a key barrier to successful CRC screening. Over half of patient participants also reported that prep is a significant barrier that could be mitigated by education about screening options. GI clinicians emphasized that navigators could fill this gap in prep education. Patients particularly noted discomfort (either experienced or reported) with liquid prep and sought out alternatives.

#### Colonoscopy logistics

Over half of partner participants identified logistics such as transportation, time off work, and cost/insurance as barriers to colonoscopy. Patients also discussed transportation or finances as barriers, though these mostly only came up when prompted by interviewers. Both patients and partners largely expressed that insurance coverage issues are less prevalent now due to improvements in coverage for various types of tests (e.g. Cologuard), though insurance came up as a potential barrier among some Latine participants.

#### Fear and stigma

Over half of patients cited fear of both a cancer diagnosis and colonoscopies as significant barriers; women made up over half of participants who cited fear of cancer diagnoses. Latine participants emphasized the role of family in the screening decision-making process to remove taboo and stigma around colonoscopy. Patients suggested that *social support* and *stool test alternatives* could alleviate fear, particularly where providers could reassure patients who have a fear of cancer that catching cancer early helps with treatment options. However, clinicians shied away from suggesting stool tests due to higher sensitivity and colonoscopy as the gold standard for CRC detection.

When asked directly, nearly all patient participants acknowledged *masculinity or sexuality* as a barrier for men considering colonoscopy when queried, including some clinicians. Black men recounted being teased for receiving a colonoscopy when telling their other male family members that they were screened, and Black women further agreed that men have stigma against colonoscopies. Latinos furthered this observation, citing embarrassment with having providers see them naked. A gay Latino participant noted that he had explained to other Latinos that a colonoscopy does not mean that a person is gay, in addition to feeling stigmatized by a provider for seeking a colonoscopy. In contrast, women did not associate colonoscopies with sexuality for themselves or other women.

When patients and clinicians were asked about potential solutions for masculinity barriers, both suggested allowing for choice in the gender of the provider. Patients also suggested normalization strategies such as explaining anesthesia and ensuring that patients understand that anyone can get CRC cancer, regardless of gender. GI partners seconded this, stating that destigmatizing colonoscopies and GI care is facilitated by trusting relationships.

### Clinic- and system-level barriers to CRC screening

Table 4 summarizes the clinic- and system-level barriers patients and providers/staff identified related to CRC screening.

**Table 4:** Clinic- and System-level barriers and navigation solutions

	Patient Quotes	Healthcare system partner quotes
Discrimination & Mistrust	Patient 4 (73 year-old Spanish speaking Latina) “But they should [do CRC screening] with the intention that if something appears, then they should tell it immediately, ***not just have it there like a little experiment mouse***,*** right?***” […] “***So I don’t see that [discrimination] in the doctors there in [home country].*** […] I’m lucky that I go to a doctor there and have a solution to the problems, right?” Patient 13 (48 year-old Black man) “A church friend of mine just had [a discrimination incident] where, ***she had some concerns***,*** and the doctor wasn’t listening to her was just backing it off***,*** as if it was a bit of hysteria***. I mean, I think it just kind of ***break down the trust***. The trust as to the information that we then receive.” Patient 9 (58 year-old English-speaking Black woman) “Yes, I feel of you break the barrier and make that person feel uncomfortable at one time [by discriminating against them], ***they either gonna find another doctor or they’re not going to come to you*** like they they’re not going to get their health checkups like they should because nobody wants to deal with that. Yeah, cuz I said I’m about to switch [doctors].” “When it comes to different people of different minorities knowing their background a little better, [sic] as opposed to putting everybody in the same boat. [sic] ***When we started the conversation***,*** and you said that Black***,*** Hispanic people are being affected by a lot***,*** [sic] that touched me because that is a true fact to me that I do know.*** When it comes to the medical field, [sic] White people get treated better, they get better treatment than us and [Latine] people. ***So when you come to me with that route***,*** then I know that you’re concerned about me and you’re speaking honestly.***”	Provider 10 (GI physician) “I’ve worked in [city] since 1998. And you know, I’ll say I medically kind of came of age, you know with some of the Kennedy Krieger history, you know vulnerable populations being exploited. ***Communities have a have a long memory***,*** you know***, I’m my parents are in their 70 s and 80 s and they, I mean they bring up the Tuskegee experiments, maybe not monthly, but regularly, you know. So I mean, considering that those just ended in the 1950 s, you know so or 60s. So there’s, ***I will say that some of that***,*** some of the mistrust fullness of the medical community***,*** those roots go deep***,*** you know.*** Also, you know perhaps in a not always knowing who’s got your best interest at heart.” Provider 6 (GI operations staff) “But I think ***there is sometimes the fear of patients of saying like***,*** Okay***,*** I don’t know***,*** my immigration status is this***,*** can I apply for insurance?*** Yes, you can, [city] allows you to. Yeah, but I think there’s sometimes that trust factor, and we’re like, please apply for insurance we need to do next steps, […] we want to treat you. So I think it’s like that understanding of like, it’s okay to update your information. We’re a health care center, we are not the police, we just want to help you.”
Clinical Workflow	Patient 2 (50 year-old Spanish-speaking Latina) ***“But that’s my doctor***,*** he sent it to me to do it again now I already made the appointment months ago***. [Now] I have to do it in March. I had an appointment in November, but they changed it for March.” Patient 9 (58 year-old Black woman) “I just went I went to the doctor, she sent me the box in the mail that you do from home and I wasn’t very happy with that. I did exactly as they asked me. And I set it outside for the man to pick it up and he came and picked it up. ***And then they wrote me back and sent me another test and they couldn’t get a reading. And they want for me to do it again.*** And I was like, No, I’m not doing it again.”	Provider 10 (GI Physician) ***“[We need to] close some of the holes when it comes to Cologuard screenings.*** If a patient has a positive Cologuard and has not gotten a referral to GI, they [need to] follow up on that. If the patient has a referral to GI, they [need to] follow up on that. If the patient has had their colonoscopy, but the primary care provider has not gotten the results and recommendations followed. They [need to] close out that hole as well. [sic] ***Unfortunately***,*** a lot of the burden falls to the patients.***” Provider 6 (GI provider) “And so ***we allow [navigators] to schedule directly on to our schedule***. So they’ll they navigate the patients, [and] I have reserved time slots on our schedule for their patients.” Provider 3 (PC physician) “***How do we capture the people in [health system] that need to get this done***,*** that aren’t necessarily coming in all the time.*** How are we dealing with that? And then I’m assuming blindly that GI has enough capacity to deal with [the navigator] sending lots of colonoscopies, which I’m sure they’re happy to do their procedures, but do they actually have the bandwidth downstream to be able to deal with it and get back in the information back to us more uniformly?” Provider 8 (Operations staff) “Yeah, well, so what tends to happen is that pre procedures, the APCs [Advanced Practice Clinicians] will go over the pre procedure instructions. ***But keep in mind***,*** their procedure might be a month or two months out.*** So when it’s coming to the day before the procedure, [the patient] is like, I just forgot everything that APC told me and I have no idea how to do this prep, or oh my gosh, I forgot to pick up my prep and prep at the pharmacy. And now it’s too late to even start what do I do so that that’s the kind of thing that we get noncompliance about.”

Emphasis added to quotes

#### Discrimination and mistrust

Patients expressed that medical mistrust could influence CRC screening, including concerns that results would not be conveyed in a timely manner. Latine patients particularly cited mistrust as relevant to CRC screening. One Latina patient recounted not trusting US-based colonoscopy processes, instead returning to her home country where the preparation is less intense for patients; others noted perceived discrimination in healthcare system encounters.

A majority of healthcare system partners did not mention barriers specific to Black or Latine patients other than language, focusing instead on barriers primarily related to socioeconomic factors (education, transportation, etc.). Notably, only a Black GI physician discussed historical racism in relation to CRC screening, bringing up historic violations of trust such as the Kennedy Krieger Institute case (conducting research with economically and educationally vulnerable children) and Tuskegee syphilis studies (depriving Black people of curative treatment) [22–24]. Another Black and Latina GI practice manager raised the issue of immigration status in relation to insurance coverage, illustrating the importance of fostering trust with immigrant communities.

For patients, suggestions to overcome discrimination and mistrust included direct communication about CRC screening and disparities in care delivery and access. Patients suggested building trust by acknowledging cultural differences and health inequities, overcoming beliefs that non-White individuals do not receive the same quality of care as White individuals.

#### Workflow and communication

All healthcare system partners identified workflow challenges that impede care, and ways for patient navigators to fill these gaps. Participants in administrative roles related to clinical operations discussed barriers to closed-loop reporting of completed stool tests and colonoscopies, including difficulty receiving colonoscopy reports from outside the healthcare system. PC physicians expressed that they may not know if a referral is completed until a subsequent appointment. Participants also cited challenges scheduling timely colonoscopies in general but especially after receipt of a positive stool test. Clinicians in PC and GI hoped that navigators could fill these gaps on communication of results and scheduling. A GI operations leader further suggested that some appointment slots could be reserved for navigated patients if there were challenges with scheduling. Partners hoped that navigators might reduce no-show rates and follow up with patients after the procedure to ensure there were no complications, as well as confirming their understanding of results. Some PC physicians further suggested that the navigator might help to identify patients who are due for CRC screening, complementing existing workflows that rely medical assistants in clinic either placing orders or reminding providers of who is due.

#### Other barriers and opportunities for improvement

One practice manager expressed that there are several competing priorities at the primary care level, and that “whoever is barking the loudest is who wins.” Thus, ensuring that leadership sees CRC screening as a priority would increase the success of a navigation program, coupled with buy-in from staff such as medical assistants. Providers and patients both emphasized that community outreach and engagement should be used to educate patients, as one-on-one education with providers or navigators alone would not be sufficient.

## Discussion

Our project soliciting multi-level perspectives revealed both anticipated and new suggestions for implementation of an at-scale community health worker navigation program to mitigate screening inequities among Black and Latine patients in our catchment area. We observed already established screening barriers for Black and Latine patients including patient knowledge, fear, mistrust, and cultural sensitivity, as well as both anticipated and new clinical considerations around workflows, relative priorities, and key clinician touchpoints to scale navigation. While some information was consistent with the literature, specific requests about patient and clinical context, including patient and organizational characteristics and perspectives, and well as the environment of available resources in our catchment area are relevant to implementing the community health worker navigation program in the parent study [13, 14]. 

Similar to previous studies, we found that a lack of knowledge, awareness of CRC risk and screening, and challenges with colonoscopy logistics were patient-identified barriers for CRC screening. A 2021 systematic review of factors influencing adherence to CRC screening cited psychological, logistical, and knowledge factors as three of the most referenced reasons for non-adherence to screening recommendations [25]. Logistics around colonoscopy scheduling, including taking time off work for patients, finding transportation, and cost/insurance factors were all brought up repeatedly in our study, which aligns with previous literature [26–29]. And previous studies demonstrate that patient navigation is a highly effective strategy to tackling patient-identified barriers, particularly among underserved populations [30–32]. 

Colonoscopy education and bowel preparation are also previously established barriers to colonoscopy screening [33–36]. Healthcare system partners saw patient navigators as trusted coaches to fill in gaps with regular clinical prep instructions, or delays in scheduling that may lead to lack of follow up. One study exploring the use of peer and professional navigators to facilitate the colonoscopy process, including guidance around bowel prep, found the overall completion rate of colonoscopy was 74.7%, a 8.7% point improvement from the healthcare system’s established adherence rate [37]. Some patients in our study suggested that because of colonoscopy prep, they would prefer stool tests; thus navigators can also be trained in educating about stool testing options when patients reject colonoscopy.

Fear, a barrier identified both in our study and in previous research, is not commonly addressed through patient navigation programs. In a systematic review, only 20% of 24 studies on CRC patient navigation programs included social and emotional support for patients [31]. Still, some research has used navigators to deliver motivational interviewing to address emotional barriers [38]. Participants in our study further suggested that open and direct conversations or including family members in conversation may be helpful overcome CRC fears.

The intersection between masculinity and CRC screening has also been previously explored, with work suggesting an association between heteronormative masculine values and completion of CRC screening [39–42], including for Black men and Latinos [39, 41, 42]. However, little research focuses on ways to mitigate this barrier. Participants in our sample noted that offering a stool test to these patients could be the best route to improving screening completion. Other participants suggested allowing for choice of their GI provider’s gender. These findings, confirmed by clinician reports, further support the call for navigators to consider culturally crafted and gender aware messaging to support CRC screening [43]. Approaches to education about CRC screening rooted in community engagement (e.g., Black men/Latinos discussing safety of CRC screening and that one is asleep during the process) may also aid in alleviating fears and removing taboos associated with CRC screening.

Patients in the present work emphasized a role for providing clear CRC screening options among adults of normal CRC risk, with several patients noting they were not aware of options other than colonoscopies for CRC screening. Test preference may be determined by several factors including patient demographics, emotional and logistical considerations, changes in recommended age at screening initiation, and prior screening experiences [44–46]. Clinicians in our sample noted a preference for colonoscopy due to it being the gold standard for screening and would be needed in the case of a positive stool test anyway [5], but also noted that they may not have time to explain this to patients during a clinic visit and thus would be willing to rely on navigators to explain options for average risk patients. The best CRC screening is the screening that patients complete, and thus navigators can be key information brokers to discuss colonoscopies with patients, and if patients do not plan to complete colonoscopy, discuss alternative screening strategies. Additionally, providing patients with options for stool testing through navigation may address waiting periods for colonoscopies, if patients were appropriate candidates [47]. 

For the Latine and Black patients in our sample, experiencing past discrimination and wariness around CRC screening was a barrier to completion. Mistrust that is rooted in past discrimination and structural harms against Black, Latine, and Indigenous people in the US is a well-documented barrier to healthcare utilization, including CRC screening [48–50]. Navigation programs that are oriented toward narrowing inequities for these groups must be aware of how to navigate these concerns rooted in past harms. Our patients identified that clear, direct, open communication including acknowledging inequities was one way to facilitate a trusting relationship with patients, similar to findings in a published study of Black screening-eligible patients [49]. Thus, tailoring navigation messaging around inequities may be a method of promoting CRC completion.

Discussing options for CRC screening between patients and providers offers an opportunity to build trust through shared decision-making and partnered care [51]. This may be of critical importance considering the common sentiment in this sample that medical mistrust is likely to impact screening. Furthermore previous work has shown that communication quality can predict CRC screening behavior [52]. Thus, PC clinicians may consider the benefits of relying on patient navigators for shared decision-making about CRC screening, given that they may not have time to complete themselves during a typical office visit. In previous navigation programs, navigators have provided education on screening options [53]. Patients in our sample further identified that direct communication about CRC risks and CRC screening, in a person’s native language at approachable literacy levels is important.

GI and PC partners in our study emphasized the role that patient navigators can play in addressing workflow barriers to CRC completion. This is a key barrier that navigation programs tend to address, by relieving clinical staff of primary communication responsibility [31]. One systematic review identified that implementation of programs to improve CRC screening in PC necessitated the engagement of the entire clinical team, leadership, and partners [54], which was also consistent with our findings. For our team to have effective program implementation, we must ensure clinical buy-in into the program from each clinic. Thus, for successful implementation of an at-scale program, programs may consider building relationships with clinical champions.

## Strengths and limitations

Strengths include that interviews were conducted in both Spanish and English, which allowed us to gather valuable insights into non-English speaking Latine patients’ preferences in the navigation program’s implementation. We also were able to capture multilevel perspectives in terms of participant roles (e.g. provider, staff, patient). There are also study limitations. First, this work was conducted out within one healthcare system in the mid-Atlantic, which may lead to geography-specific concerns. However, our healthcare system incorporates diverse clinics in terms of geographic and patient characteristics, thus increasing generalizability to other health systems. Similarly, despite attempts to recruit a never-screened population, the majority of participants received CRC screening in the past. This is largely due to inconsistency in the way patients would be identified as “due” in their chart and lack of consistency in or availability of reporting up-to-date information. However, this was an important finding insofar as it informed the research team about limitations of the EHR in identifying CRC screening completion. Regardless, future work may consider how to reach people who have no prior CRC screening experience to target unique barriers they may face.

Lastly, our interview guide was developed carefully with feedback from our community advisory board, however some questions may be considered “leading” – for example, our masculinity question states that other people have connected colonoscopy to sexuality. Given the sensitive nature of this topic, we opted to normalize this issue to allow patients to feel comfortable disclosing their views. Though we chose to be responsive to this suggestion from our advisory board to ask directly about the association, others may choose a more open-ended approach.

## Conclusions

CRC burden in the US is inequitable for Black and Latine people, and timely screening is a key factor to mitigate differences in outcomes. Patient navigation is a tool to improve CRC screening among all populations, but should be culturally competent and address barriers specific to populations with lower screening rates or additional barriers to care. The current work is informing the implementation of patient navigation within our healthcare system [15]. Our work highlights that patient navigators should communicate in a direct manner to foster a trusting relationship, offer education about CRC screening options, frame CRC screening around inequities, and be prepared to navigate patients around typical CRC screening barriers. Additionally, embedding navigation within PC clinics will be beneficial for communication, and buy-in from clinical partners will be key to implementing a system-level navigation program.

## Data Availability

The datasets generated and/or analyzed during the current study are not publicly available due to protection of participant confidentiality but are available from the corresponding author on reasonable request.

## References

[1] Siegel RL, Wagle NS, Cercek A, Smith RA, Jemal A. Colorectal cancer statistics, 2023. Cancer J Clin. 2023;73(3):233–54. 10.3322/caac.21772 36856579

[2] Colorectal Cancer Facts & Figs. 2023–2025. Atlanta: American Cancer Society; 2023.

[3] Cancer Facts and. Figures for hispanic/latino people 2024–2026. Atlanta: American Cancer Society; 2024.

[4] Zheng S, Schrijvers JJA, Greuter MJW, Kats-Ugurlu G, Lu W, de Bock GH. Effectiveness of Colorectal Cancer (CRC) screening on all-cause and CRC-specific mortality reduction: a systematic review and meta-analysis. Cancers (Basel). 2023;15(7). 10.3390/cancers15071948. 10.3390/cancers15071948 PMC10093633 37046609

[5] Force UPST. Screening for colorectal cancer: US preventive services task force recommendation statement. JAMA. 2021;325(19):1965–77. 34003218 10.1001/jama.2021.6238

[6] May FP, Yang L, Corona E, Glenn BA, Bastani R. Disparities in colorectal Cancer screening in the united States before and after implementation of the affordable care act. Clin Gastroenterol Hepatol. 2020;18(8):1796–e8042. 31525514 10.1016/j.cgh.2019.09.008

[7] United States Cancer Statistics: data visualizations. Centers for Disease Control and Prevention. 2023. Available from: https://www.cdc.gov/cancer/dataviz. Cited 21 Apr 2025.

[8] Sly JR, Edwards T, Shelton RC, Jandorf L. Identifying barriers to colonoscopy screening for nonadherent African American participants in a patient navigation intervention. Health Educ Behav. 2013;40(4):449–57. 23086556 10.1177/1090198112459514 PMC3695062

[9] Byrd TL, Calderón-Mora J, Salaiz R, Shokar NK. Barriers and facilitators to colorectal Cancer screening within a Hispanic population. Hispanic Health Care Int. 2019;17(1):23–9. 10.1177/1540415318818982 30574791

[10] Farr DE, Haynes VE, Armstead CA, Brandt HM. Stakeholder perspectives on colonoscopy navigation and colorectal Cancer screening inequities. J Cancer Educ. 2021;36(4):670–6. 31970699 10.1007/s13187-019-01684-2

[11] DeGroff A, Coa K, Morrissey KG, Rohan E, Slotman B. Key considerations in designing a patient navigation program for colorectal cancer screening. Health Promot Pract. 2014;15(4):483–95. 24357862 10.1177/1524839913513587 PMC4618321

[12] Okasako-Schmucker DL, Peng Y, Cobb J, Buchanan LR, Xiong KZ, Mercer SL, et al. Community health workers to increase cancer screening: 3 community guide systematic reviews. Am J Prev Med. 2022:579–94. 10.1016/j.amepre.2022.10.016. 10.1016/j.amepre.2022.10.016 PMC10033345 36543699

[13] Feldstein AC, Glasgow RE. A practical, robust implementation and sustainability model (PRISM) for integrating research findings into practice. Joint Comm J Qual Patient Saf. 2008;34(4):228–43. 10.1016/s1553-7250(08)34030-6 18468362

[14] McCreight MS, Rabin BA, Glasgow RE, Ayele RA, Leonard CA, Gilmartin HM, et al. Using the practical, robust implementation and sustainability model (PRISM) to qualitatively assess multilevel contextual factors to help plan, implement, evaluate, and disseminate health services programs. Translational Behav Med. 2019;9(6):1002–11. 10.1093/tbm/ibz085 31170296

[15] Rivera Rivera JN, AuBuchon KE, Schubel LC, Starling C, Tran J, Locke M, et al. Supporting colorectal equitable navigation (SCREEN): a protocol for a stepped-wedge cluster randomized trial for patient navigation in primary care. Implement Sci Commun. 2024;5(1):60. 38831365 10.1186/s43058-024-00598-5 PMC11149321

[16] Creswell JW, Poth CN. Qualitative inquiry and research design: Choosing among five approaches. 5th ed. Sage; 2016.

[17] Tong A, Sainsbury P, Craig J. Consolidated criteria for reporting qualitative research (COREQ): a 32-item checklist for interviews and focus groups. Int J Qual Health Care. 2007;19(6):349–57. 17872937 10.1093/intqhc/mzm042

[18] Berger R. Now I see it, now I don’t: researcher’s position and reflexivity in qualitative research. Qualitative Res. 2015;15(2):219–34.

[19] Bourke B, Positionality. Reflecting on the research process. Qualitative Rep. 2014;19(33):1–9.

[20] Olmos-Vega FM, Stalmeijer RE, Varpio L, Kahlke R. A practical guide to reflexivity in qualitative research: AMEE guide 149. Med Teach. 2023;45(3):241–51. 10.1080/0142159X.2022.2057287 35389310

[21] Rankl F, Johnson GA, Vindrola-Padros C. Examining what we know in relation to how we know it: A team-based reflexivity model for rapid qualitative health research. Qual Health Res. 2021;31(7):1358–70. 33745367 10.1177/1049732321998062 PMC8182295

[22] Heintzelman CA. The Tuskegee syphilis study and its implications for the 21st century. New Social Worker. 2003;10(4):4–5.

[23] Shamoo AE. Ethically questionable research with children: the Kennedy krieger lead abatement study. Account Research: Policies Qual Assur. 2002;9(3–4):167–75. 10.1080/08989620214684 12816125

[24] The Untreated Syphilis Study at Tuskegee Timeline Atlanta. Centers for Disease Control and Prevention. Available from: https://www.cdc.gov/tuskegee/about/timeline.html.

[25] Dressler J, Johnsen A, Madsen LJ, Rasmussen M, Jorgensen L. Factors affecting patient adherence to publicly funded colorectal cancer screening programmes: a systematic review. Public Health. 2021;190:67–74. 33360029 10.1016/j.puhe.2020.10.025

[26] Yearby R. Racial disparities in health status and access to healthcare: the continuation of inequality in the united States due to structural racism. Am J Econ Sociol. 2018;77(3–4):1113–52.

[27] Ortega AN, Roby DH. Ending structural racism in the US health care system to eliminate health care inequities. JAMA. 2021;326(7):613–5. 34402851 10.1001/jama.2021.11160

[28] White PM, Itzkowitz SH. Barriers driving Racial disparities in colorectal Cancer screening in African Americans. Curr Gastroenterol Rep. 2020;22(8):41. 32647903 10.1007/s11894-020-00776-0

[29] Kane WJ, Fleming MA 2nd, Lynch KT, Friel CM, Williams MD, Hedrick TL, et al. Associations of race, ethnicity, and social determinants of health with colorectal Cancer screening. Dis Colon Rectum. 2023;66(9):1223–33. 35533321 10.1097/DCR.0000000000002371 PMC9643677

[30] Reuland DS, Brenner AT, Hoffman R, McWilliams A, Rhyne RL, Getrich C, et al. Effect of combined patient decision aid and patient navigation vs usual care for colorectal cancer screening in a vulnerable patient population: a randomized clinical trial. JAMA Intern Med. 2017;177(7):967–74. 28505217 10.1001/jamainternmed.2017.1294 PMC5710456

[31] Mosquera I, Todd A, Balaj M, Zhang L, Benitez Majano S, Mensah K, et al. Components and effectiveness of patient navigation programmes to increase participation to breast, cervical and colorectal cancer screening: a systematic review. Cancer Med. 2023;12(13):14584–611. 37245225 10.1002/cam4.6050 PMC10358261

[32] Winkler CS, Hardaway JC, Ceyhan ME, Espat NJ, Saied Calvino A. Decreasing colorectal cancer screening disparities: A culturally tailored patient navigation program for Hispanic patients. Cancer. 2022;128(9):1820–5. 35128638 10.1002/cncr.34112

[33] Jones RM, Devers KJ, Kuzel AJ, Woolf SH. Patient-reported barriers to colorectal cancer screening: a mixed-methods analysis. Am J Prev Med. 2010;38(5):508–16. 20409499 10.1016/j.amepre.2010.01.021 PMC2946825

[34] Travis E, Kerrison RS, O’Connor DB, Ashley L. Barriers and facilitators to colonoscopy for cancer detection: patient and practitioner perspectives. Psychol Health. 2024;39(9):1263–83. 36373225 10.1080/08870446.2022.2141241

[35] Kerrison RS, Sheik-Mohamud D, McBride E, Whitaker KL, Rees C, Duffy S, et al. Patient barriers and facilitators of colonoscopy use: A rapid systematic review and thematic synthesis of the qualitative literature. Prev Med. 2021;145:106413. 33412167 10.1016/j.ypmed.2020.106413

[36] McLachlan S-A, Clements A, Austoker J. Patients’ experiences and reported barriers to colonoscopy in the screening context—a systematic review of the literature. Patient Educ Couns. 2012;86(2):137–46. 21640543 10.1016/j.pec.2011.04.010

[37] Jandorf L, Cooperman JL, Stossel LM, Itzkowitz S, Thompson HS, Villagra C, et al. Implementation of culturally targeted patient navigation system for screening colonoscopy in a direct referral system. Health Educ Res. 2013;28(5):803–15. 23393099 10.1093/her/cyt003 PMC3772329

[38] Temucin E, Nahcivan NO. The effects of the nurse navigation program in promoting colorectal Cancer screening behaviors: a randomized controlled trial. J Cancer Educ. 2020;35(1):112–24. 30470978 10.1007/s13187-018-1448-z

[39] Rogers CR, Perdue DG, Boucher K, Korous KM, Brooks E, Petersen E, et al. Masculinity barriers to ever completing colorectal cancer screening among American indian/alaska native, black, and white men (ages 45–75). IJERPH. 2022;19(5):3071. 35270762 10.3390/ijerph19053071 PMC8910566

[40] Sedani AE, Islam JY, Griffith DM, Rifelj KK, McCall C, García-Rodríguez O, et al. Sociocultural and masculinity influences on colorectal cancer screening participation among hispanic/latino men in florida, new york, and Texas. Cancer Med. 2024;13(18):e70159. 39302027 10.1002/cam4.70159 PMC11413917

[41] Rogers CR, Rogers TN, Matthews P, Le Duc N, Zickmund S, Powell W, et al. Psychosocial determinants of colorectal Cancer screening uptake among African-American men: Understanding the role of masculine role norms, medical mistrust, and normative support. Ethn Health. 2022;27(5):1103–22. 33249920 10.1080/13557858.2020.1849569 PMC8163893

[42] Christy SM, Mosher CE, Rawl SM. Integrating men’s health and masculinity theories to explain colorectal cancer screening behavior. Am J Mens Health. 2014;8(1):54–65. 23813927 10.1177/1557988313492171 PMC3849215

[43] Lucas T, Rogers CR, Aspiras O, Manning M, Dawadi A, Thompson HS. Message framing for men? Gender moderated effects of culturally targeted message framing on colorectal cancer screening receptivity among African Americans. Psychol Men Masculinities. 2023;24(2):103. 10.1037/men0000418 PMC10181814 37193560

[44] Lee SJ, O’Leary MC, Umble KE, Wheeler SB. Eliciting vulnerable patients’ preferences regarding colorectal cancer screening: a systematic review. Patient Prefer Adherence. 2018:2267–82. 10.2147/PPA.S156552. 10.2147/PPA.S156552 PMC6216965 30464417

[45] Makaroff KE, Shergill J, Lauzon M, Khalil C, Ahluwalia SC, Spiegel BM, et al. Patient preferences for colorectal cancer screening tests in light of Lowering the screening age to 45 years. Clin Gastroenterol Hepatol. 2023;21(2):520–31. e10. 35870766 10.1016/j.cgh.2022.07.012 PMC9852355

[46] Heidenreich S, Finney Rutten LJ, Miller-Wilson LA, Jimenez‐Moreno C, Chua GN, Fisher DA. Colorectal cancer screening preferences among physicians and individuals at average risk: A discrete choice experiment. Cancer Med. 2022;11(16):3156–67. 35315224 10.1002/cam4.4678 PMC9385595

[47] Coronado GD, Bienen L, Burnett-Hartman A, Lee JK, Rutter CM. Maximizing scarce colonoscopy resources: the crucial role of stool-based tests. JNCI: J Natl Cancer Inst. 2024;116(5):647–52. 38310359 10.1093/jnci/djae022 PMC11491837

[48] Taylor LA, Nong P, Platt J. Fifty years of trust research in health care: a synthetic review. Milbank Q. 2023;101(1):126–78. 36689251 10.1111/1468-0009.12598 PMC10037697

[49] Aspiras O, Lucas T, Thompson HS, Manning MA. Medical mistrust, culturally targeted message framing, and colorectal cancer screening among African Americans. J Behav Med. 2023;46(5):871–81. 37140761 10.1007/s10865-023-00415-9

[50] Bazargan M, Cobb S, Assari S. Discrimination and medical mistrust in a Racially and ethnically diverse sample of California adults. Annals Family Med. 2021;19(1):4–15. 10.1370/afm.2632 PMC7800756 33431385

[51] Hong H, Oh HJ. The effects of patient-centered communication: exploring the mediating role of trust in healthcare providers. Health Commun. 2020;35(4):502–11. 30706741 10.1080/10410236.2019.1570427

[52] Scott AM, Van Scoy LJ, Chinchilli VM, Ruffin MT, Wasserman E, Jimbo M. Communication quality predicts patients’ colorectal cancer screening behavior. Soc Sci Med. 2024;358:117199. 39168066 10.1016/j.socscimed.2024.117199

[53] Muliira JK, D’Souza MS. Effectiveness of patient navigator interventions on uptake of colorectal cancer screening in primary care settings. Japan J Nurs Sci. 2016;13(2):205–19. 26543010 10.1111/jjns.12102

[54] Adhikari K, Manalili K, Law J, Bischoff M, Teare GF. Interventions to increase colorectal Cancer screening uptake in primary care: A systematic review. J Am Board Fam Med. 2022;35(4):840–58. 35896448 10.3122/jabfm.2022.04.210399

